# Vestibular Dysfunction and Difficulty with Driving: Data from the 2001–2004 National Health and Nutrition Examination Surveys

**DOI:** 10.3389/fneur.2017.00557

**Published:** 2017-10-17

**Authors:** Eric X. Wei, Yuri Agrawal

**Affiliations:** ^1^Department of Otolaryngology-Head and Neck Surgery, Johns Hopkins University School of Medicine, Baltimore, MD, United States

**Keywords:** vestibular system, spatial cognition, driving, falls, aging

## Abstract

**Background and objective:**

There is growing understanding of the role of vestibular function in spatial navigation and orientation. Individuals with vestibular dysfunction demonstrate impaired performance on static and dynamic tests of spatial cognition, but there is sparse literature characterizing how these impairments might affect individuals in the real-world. Given the important role of visuospatial ability in driving a motor vehicle, we sought to evaluate whether individuals with vestibular dysfunction might have increased driving difficulty.

**Materials and methods:**

We used data from the 2001–2004 National Health and Nutrition Examination Surveys to evaluate the influence of vestibular dysfunction in driving difficulty in a nationally representative sample of U.S. adults aged ≥50 years (*n* = 3,071). Vestibular function was measured with the modified Romberg test. Furthermore, since vestibular dysfunction is a known contributor to falls risk, we assessed whether individuals with vestibular dysfunction and concomitant driving difficulty were at an increased risk of falls.

**Results:**

In multivariate analyses, vestibular dysfunction was associated with a twofold increased odd of driving difficulty (odds ratio 2.16, 95% CI 1.57, 2.98). Among participants with vestibular dysfunction, concomitant driving difficulty predicted an increased risk of falls that was significantly higher than in participants with vestibular dysfunction only (odds ratio 13.01 vs. 2.91, *p* < 0.0001).

**Conclusion:**

This study suggests that difficulty driving may be a real-world manifestation of impaired spatial cognition associated with vestibular loss. Moreover, driving difficulty may be a marker of more severe vestibular dysfunction.

## Introduction

An emerging body of evidence suggests that vestibular function is critical for spatial orientation and navigation (1–4). One recent study in community-dwelling adults found a significant association between vestibular function and tests of visuospatial cognitive ability (4). Studies using the triangle completion task (TCT) have shown that vestibular function (both otolith and canal) contributes to performance on a dynamic spatial navigation task (5, 6). Furthermore, patients with bilateral vestibular loss were shown to have lower hippocampal volumes and impaired spatial memory and navigation as assessed with a virtual maze test (7). Despite the numerous lines of evidence linking vestibular loss to impairments in spatial cognition, there is a limited amount of literature demonstrating the real-world consequences of impaired spatial cognition in patients with vestibular loss.

Driving a motor vehicle is one task that appears to reflect spatial cognitive ability (8–12). Neuropsychological studies have identified that spatial cognition as determined by visuospatial tests such as the Intersecting Pentagon Copying, Clock-Face Drawing, and Block Design tests are predictive of on-road driving test performance (9, 12). Older adults in particular experience greater driving errors and crash involvement, which has been associated with declining visuospatial ability with age (13–16). Some evidence has emerged reporting driving difficulty in patients with vestibular disorders such as Meniere’s disease, BPPV, chronic vestibulopathy, and patients with postoperative acoustic neuromas resection or vestibular nerve section (17–20). However, there are limited data on whether vestibular loss in the general population of older adults may be associated with the greater difficulties in driving ability that occur with age.

In this study of data from the 2001–2004 National Health and Nutrition Examination Surveys (NHANES), we evaluated whether vestibular dysfunction, as assessed by performance on a postural metric, was associated with self-reported driving difficulty in participants aged 50 years and older. Furthermore, given that vestibular dysfunction is a significant contributor to falls risk, we evaluated whether driving difficulty may indicate an increased severity of vestibular loss associated with increased fall risk. These analyses demonstrate a real-world behavioral correlate of the role of vestibular function in spatial navigation and further provide insights into the clinical management of patients with vestibular impairments and concomitant driving difficulty.

## Materials and Methods

### Study Population

National Health and Nutrition Examination Surveys is an ongoing cross-sectional survey of the civilian, non-institutionalized population of the USA conducted by the Centers for Disease Control and Prevention. Every 2 years, NHANES enrolls randomly selected participants for a comprehensive health screening, creating a nationally representative sample. The response rate was 84 and 79% in the 2001–2002 and 2003–2004 cycles, respectively (21). Further details of the NHANES sampling process have been published previously (22, 23).

The 2001–2002 and 2003–2004 NHANES performed balanced testing and queried about difficulty driving in a nationally representative sample of adults aged 50 years and older. We combined these two 2-year cycles of data to analyze 4 years of data, per National Center for Health Statistics (NCHS) recommendations (22). A total of 21,161 individuals of all ages took part in NHANES from 2001 to 2004; 5,073 individuals (24.0%) were 50 years or older. Participants were excluded from balance testing if they were unable to stand on their own, were having dizziness or lightheadedness sufficient to cause unsteadiness, weighed more than 124.7 kg (275 pounds), had a waist circumference that could not accommodate proper fitting of the standard-sized safety gait belt, needed a leg brace to stand unassisted, or had a foot or leg amputation. In addition, participants who were totally blind or sufficiently visually impaired to require assistance in finding the examination room were excluded from participation. A total of 453 participants (8.9%) were excluded because of these reasons, yielding an eligible sample of 4,620 participants. Of these eligible adults, 906 participants (21.3%) were excluded because they did not participate in the NHANES physical examination for various reasons including “safety exclusion” and “participant refusal,” resulting in 3,714 participants (78.7% of eligible participants). Included participants were more likely to be younger and white. Sample weights for the combined 4-year sample were used per NCHS guidelines (23).

This study was carried out in accordance with the recommendations of National Center for Health Statistics Research Ethics Review Board with written informed consent from all subjects. All subjects gave written informed consent in accordance with the Declaration of Helsinki. The protocol was approved by the National Center for Health Statistics Research Ethics Review Board.

### Balance Testing

Balance testing consisted of the modified Romberg Test of Standing Balance on Firm and Compliant Support Surfaces. This test examined the participant’s ability to stand unassisted using four test conditions. These conditions were ordered in increasing level of difficulty and were designed specifically to test the sensory inputs that contribute to balance, specifically the vestibular system, vision, and proprioception. The fourth test condition was designed to test vestibular function exclusively: participants had to maintain balance on a foam-padded surface (to obscure proprioceptive input) with their eyes closed (to eliminate visual input), thereby relying exclusively on vestibular input to maintain upright stance.

Each of the four balance test conditions was assessed on a pass/fail basis. Test failure was defined as participants needing to open their eyes; moving their arms or feet to achieve stability; or beginning to fall or requiring operator intervention to maintain balance within a 15-s interval (test conditions 1 and 2) or a 30-s interval (test conditions 3 and 4). Each participant who failed a test condition was eligible for 1 retest. The protocol for retesting was the same as for the primary examination. Because each successive test condition was more difficult than the condition preceding it, balance testing was concluded whenever a subject failed to pass a test condition (during the initial test or in the retest). We focused on test condition 4—standing with eyes closed on a 40.6 cm × 45.7 cm × 7.6 cm (16″ × 18″ × 3″) foam pad—in which participants relied primarily on vestibular input for balance. We categorized participants as having vestibular dysfunction if they did not pass test condition 4. Of the 3,714 participants, 245 (6.6%) participants did not pass prior test conditions and thus did not participate in test condition 4. An additional 78 (2.1%) participants had missing data for test condition four, 252 (6.8%) participants had missing questionnaire data, 66 (1.8%) participants had missing visual acuity testing data, and two (0.1%) participants had missing demographic data, leading to a total of 643 excluded participants (17.3%), yielding a final sample size of 3,071. Among the 3,071 participants, 1,594 (51.9%) participants did not pass test condition 4. For those who did not pass test condition 4, failure time, ranging from 0 to 29 s, was averaged between the test and retest. 30 (1.9%) participants who did not pass test condition 4 had missing or incomplete failure time data. Further details of balance testing procedures are available at http://www.cdc.gov/nchs/data/nhanes/ba.pdf.

### Questionnaire

Trained interviewers administered detailed questionnaires prior to balance testing. The study population was stratified into age groups (50–59, 60–69, 70–79, and older than 80 years). Race-ethnicity was grouped as non-Hispanic white (hereafter, “white”), non-Hispanic black (hereafter, “black”), Mexican American, or others. Education was grouped as less than high school, high school diploma (including GED), or beyond high school.

Participants were asked about a history of dizziness (“During the past 12 months, have you had dizziness or difficulty with balance?”) and falls (“During the past 12 months, have you had difficulty with falling?”). Participants were also queried about any difficulty with driving (“How much difficulty do you have driving during the daytime in familiar places?”). Among 3,071 participants, 2,792 (90.9%) participants had no difficulty, 69 (2.3%) participants had a little difficulty, 15 (0.5%) participants had moderate difficulty, 6 (0.2%) participants had extreme difficulty, 36 (1.2%) participants were unable to drive because of eyesight, and 153 (5.0%) participants were unable to drive because of other reasons. Participants were excluded if they never drove, responded “do not know,” or refused the question. Responses were dichotomized into “no difficulty” versus “any difficulty” in line with previous studies (24, 25).

### Visual Acuity Assessment

We also considered visual activity given that it contributes to driving ability (26, 27). Presenting distance visual acuity was measured in each eye using an autorefractor containing built-in visual acuity charts (Nidek ARK-760, Tokyo, Japan) with whichever form of correction (e.g., glasses and contact lenses), if any, that the participant was wearing (or brought with them) to view distant objects on the day they visited the Mobile Examination Center. Presenting distance visual acuity was recorded as the smallest line for which at least four out of five characters were read correctly. Distance visual acuity was then re-measured with the autorefractor correction in place for all eyes for which presenting visual acuity was deemed to be 20/30 or worse.

Presenting visual acuity impairment was defined as a presenting visual acuity of worse than 20/40 in the better-seeing eye. Uncorrectable visual acuity impairment was defined as having a visual acuity of worse than 20/40 in the better-seeing eye after autocorrection. Correctable visual acuity impairment was defined as having a presenting visual acuity of worse than 20/40 that improved to 20/40 or better with autocorrection.

### Analyses

The main outcome of interest was difficulty with driving. The predictor variables were vestibular dysfunction and demographic variables. We estimated the prevalence of difficulty with driving in the overall population and stratified by demographic characteristics. The χ^2^ *F* statistic was used to test for overall differences in proportions. Multiple logistic regression was used to estimate the odds of experiencing driving difficulty associated with vestibular dysfunction, visual acuity, and demographic characteristics. Multiple logistic regression analyses were further used to estimate the odds of falling associated with driving difficulty, vestibular dysfunction, visual acuity, and demographic characteristics.

All analyses were adjusted for the survey design using the SVY procedures in Stata software (StataCorp, College Station, TX, USA). Sample weights were incorporated into all analyses by using the pweight statement in Stata software per NCHS instructions. All coefficients, odds ratios, and variance estimates are presented from weighted analyses unless otherwise specified. *p* Values less than 0.05 were considered statistically significant.

## Results

Difficulty with driving was reported by 9.1% of participants aged 50 years and older from the 2001–2004 NHANES (Table 1). There were significant differences in the prevalence of driving difficulty by sex, age, race/ethnicity, education, presenting visual acuity, vestibular function, and history of falls. The prevalence of driving difficulty increased markedly with age and was higher in females. There was a higher prevalence of driving difficulty among blacks, Mexican-Americans, and participants in the “other” race/ethnicity category compared to whites. There was also a lower prevalence of driving difficulty among individuals with a high school education or beyond a high school education compared to those with a less than high school education. Individuals with presenting visual acuity impairment, either correctable or uncorrectable, experienced driving difficulty at higher rates. Individuals with vestibular dysfunction and a history of falls within the past year reported higher rates of driving difficulty.

**Table 1:** Characteristics of study sample by driving difficulty status, National Health and Nutrition Examination Surveys 2001–2004.

	*N* = 2,934		
	No difficulty with driving *N* = 2,840 *N* (%)	Difficulty with driving *N* = 95 *N* (%)	*p*-Value
**Sex**			**0.0013**
Male	1,506 (53.9%)	121 (43.4%)	
Female	1,286 (46.1%)	158 (56.6%)	
**Age (years)**			**<0.0001**
50–59	887 (31.8%)	47 (16.9%)	
60–69	981 (35.1%)	64 (22.9%)	
70–79	635 (22.7%)	83 (29.8%)	
80 and above	2,289 (10.4%)	85 (30.5%)	
**Race**			**<0.0001**
Non-Hispanic White	1,833 (65.7%)	144 (51.2%)	
Non-Hispanic Black	412 (14.8%)	62 (22.2%)	
Mexican American	409 (14.7%)	51 (18.3%)	
Other race	138 (4.9%)	22 (7.9%)	
**Education**			**<0.0001**
Less than high school	713 (25.5%)	145 (51.9%)	
High school	691 (24.8%)	58 (20.8%)	
Beyond high school	1,388 (49.7%)	76 (27.2%)	
**Presenting visual acuity**			**<0.0001**
Without impairment	2,614 (93.6%)	206 (73.8%)	
Correctable impairment	117 (4.2%)	28 (10.0%)	
Uncorrectable impairment	61 (2.2%)	45 (16.1%)	
**Vestibular function**			**<0.0001**
Normal	1,407 (50.4%)	70 (25.1%)	
Dysfunction	1,385 (49.6%)	209 (74.9%)	
**Falls within past year^a^**			**<0.0001**
Yes	2,693 (96.5%)	239 (85.7%)	
No	98 (3.5%)	40 (14.3%)	

*^a^Missing data on one participant, who responded “do not know” to question on falls*.

*Bolded p-values were considered significant (p < 0.05)*.

Multiple logistic regression analyses were conducted to assess the association between vestibular dysfunction and driving difficulty, after adjusting for potential confounders (Table 2). Relative to participants with normal vestibular function, individuals with vestibular dysfunction had a significantly increased odds of driving difficulty (odds ratio 2.16, 95% confidence interval 1.57, 2.98). We further evaluated the odds of driving difficulty among participants with vestibular dysfunction who were clinically symptomatic (as determined by self-reported difficulty with dizziness or balance). We found that these participants had a greater than fourfold increase in the odds of driving difficulty (odds ratio 4.30, 95% confidence interval 2.73, 6.78) compared to participants without vestibular dysfunction in adjusted analyses (data not shown). Notably, participants with vestibular dysfunction who were not clinically symptomatic also had a significant increased odds of driving difficulty (odds ratio 1.47, 95% confidence interval 1.05, 2.06) compared to participants without vestibular dysfunction in adjusted analyses (data not shown). Among participants who did not pass test condition 4 (*N* = 1,564), each additional second of maintaining balance before test failure was associated with a significantly decreased odds of driving difficulty (odds ratio 0.93, 95% confidence interval 0.87, 0.99) in multiple logistic regression (data not shown).

**Table 2:** Vestibular dysfunction is associated with driving difficulty, National Health and Nutrition Examination Surveys 2001–2004.

	Odds ratio (95% CI)	*p*-Value
Vestibular dysfunction	2.16 (1.57, 2.98)	**<0.0001**
**Sex**		
Male	1.0 (reference)	
Female	1.87 (1.32, 2.66)	**0.0010**
**Age (years)**		
50–59	1.0 (reference)	
60–69	0.94 (0.57, 1.56)	0.80
70–79	1.98 (1.35, 2.91)	**0.0009**
80 and above	4.56 (2.55, 8.13)	**<0.0001**
**Race**		
Non-Hispanic White	1.0 (reference)	
Non-Hispanic Black	3.04 (2.01, 4.60)	**<0.0001**
Mexican American	1.72 (0.92, 3.19)	0.085
Other race	3.17 (1.78, 5.66)	**0.0003**
**Education**		
Less than high school	1.0 (reference)	
High school	0.40 (0.26, 0.61)	**0.0001**
Beyond high school	0.25 (0.17, 0.38)	**<0.0001**
**Presenting visual acuity**		
Without impairment	1.0 (reference)	
Correctable impairment	1.51 (0.99, 2.31)	0.055
Uncorrectable impairment	3.99 (2.04, 7.81)	**0.0002**

*Bolded p-values were considered significant (p **<** 0.05)*.

We further explored whether driving difficulty may be associated with falls risk and evaluated the relationship between vestibular dysfunction and both driving and falls (Table 3). We found that individuals with driving difficulty had an increased odds of falling (odds ratio 5.38, 95% confidence interval 2.94, 9.84) in multiple logistic regression. To explore whether the relationship between driving difficulty and falls could be explained by their shared association with vestibular dysfunction (i.e., vestibular dysfunction is a *confounder* of the association between driving difficulty and falls), we adjusted for vestibular dysfunction in multiple logistic regression analysis. After adjusting for vestibular dysfunction, the odds of falling associated with driving difficulty were slightly attenuated (odds ratio 4.77, 95% confidence interval 2.67, 8.53), while vestibular dysfunction was independently associated with an increased odds of falls (odds ratio 2.77, 95% confidence interval 1.50, 5.12). This analysis suggests that vestibular dysfunction is a partial confounder of the association between driving difficulty and falls.

**Table 3:** Vestibular dysfunction mediates the association between driving difficulty and falls, National Health and Nutrition Examination Surveys 2001–2004.

	Model without vestibular dysfunction		Model with vestibular dysfunction	
	Odds ratio (95% CI)	*p*-Value	Odds ratio (95% CI)	*p*-Value
Vestibular dysfunction	**–**	**–**	2.77 (1.50, 5.12)	**0.002**
Driving difficulty	5.38 (2.94, 9.84)	**<0.0001**	4.77 (2.67, 8.53)	**<0.0001**
**Sex**				
Male	1.0 (reference)		1.0 (reference)	
Female	1.92 (1.23, 3.00)	**0.005**	1.94 (1.26, 3.00)	**0.004**
**Age (years)**				
50–59	1.0 (reference)		1.0 (reference)	
60–69	1.16 (0.57, 2.35)	0.67	0.99 (0.47, 2.09)	0.97
70–79	1.42 (0.67, 3.05)	0.35	1.04 (0.44, 2.42)	0.93
80 and above	1.41 (0.73, 2.75)	0.30	0.91 (0.45, 1.82)	0.78
**Race**				
Non-Hispanic White	1.0 (reference)		1.0 (reference)	
Non-Hispanic Black	0.73 (0.39, 1.38)	0.32	0.77 (0.40, 1.47)	0.41
Mexican American	0.64 (0.36, 1.16)	0.13	0.60 (0.32, 1.11)	0.10
Other race	1.34 (0.54, 3.33)	0.52	1.22 (0.47, 3.18)	0.68
**Education**				
Less than high school	1.0 (reference)		1.0 (reference)	
High school	1.04 (0.67, 1.62)	0.87	1.05 (0.66, 1.69)	0.83
Beyond high school	0.72 (0.41, 1.26)	0.24	0.77 (0.44, 1.34)	0.34
**Presenting visual acuity**				
Without impairment	1.0 (reference)		1.0 (reference)	
Correctable impairment	1.18 (0.54, 2.60)	0.67	1.16 (0.52, 2.59)	0.71
Uncorrectable impairment	0.83 (0.26, 2.64)	0.74	0.83 (0.26, 2.69)	0.75

*Bolded p-values were considered significant (p **<** 0.05)*.

We compared the odds of falling between individuals with vestibular dysfunction with and without associated difficulty driving (Table 4). We found that participants with vestibular dysfunction without driving difficulty had a nearly threefold increased odds of falling (odds ratio 2.91, 95% confidence interval 1.61, 5.26). Meanwhile, individuals with both vestibular dysfunction and difficulty driving had an over 13-fold increase in the odds of falling (odds ratio 13.01, 95% confidence interval 4.95, 32.19) relative to participants with neither driving difficulty nor vestibular dysfunction in adjusted analyses. According to postestimation adjusted Wald test, individuals with both vestibular dysfunction and driving difficulty had a significantly higher odds of falling as compared to individuals with only vestibular dysfunction (*p* < 0.0001).

**Table 4:** Vestibular dysfunction and concomitant driving difficulty on odds of falls, National Health and Nutrition Examination Surveys 2001–2004.

	Odds ratio (95% CI)	*p*-Value
**Vestibular and/or driving impairment**		
Neither impairments	1.0 (reference)	
Vestibular dysfunction only	2.91 (1.61, 5.26)	**0.0009**
Driving difficulty only	6.20 (2.48, 15.52)	**0.0003**
Both vestibular and driving impairments	13.01 (4.95, 34.19)	**<0.0001**
**Sex**		
Male	1.0 (reference)	
Female	1.95 (1.26, 3.01)	**0.004**
**Age (years)**		
50–59	1.0 (reference)	
60–69	0.98 (0.47, 2.08)	0.97
70–79	1.04 (0.44, 2.42)	0.93
80 and above	0.92 (0.46, 1.84)	0.81
**Race**		
Non-Hispanic White	1.0 (reference)	
Non-Hispanic Black	0.76 (0.40, 1.42)	0.37
Mexican American	0.60 (0.32, 1.11)	0.10
Other race	1.22 (0.46, 3.21)	0.68
**Education**		
Less than high school	1.0 (reference)	
High school	1.05 (0.66, 1.68)	0.83
Beyond high school	0.77 (0.44, 1.33)	0.34
**Presenting visual acuity**		
Without impairment	1.0 (reference)	
Correctable impairment	1.17 (0.53, 2.58)	0.68
Uncorrectable impairment	0.84 (0.26, 2.68)	0.76

*Bolded p-values were considered significant (p **<** 0.05)*.

## Discussion

In this analysis of a nationally representative sample, vestibular dysfunction, as defined by impaired performance on a postural metric, was significantly associated with difficulty driving a motor vehicle. Participants with vestibular dysfunction who were clinically symptomatic experienced a fourfold increased odd of reporting difficulty with driving. Notably, individuals with vestibular dysfunction who were asymptomatic (i.e., subclinical vestibular dysfunction) were also at significantly increased odds of experiencing driving difficulty. Among individuals with vestibular dysfunction, concomitant driving difficulty predicted a significantly increased risk of falls compared to individuals with vestibular dysfunction without driving difficulty. These findings support the hypothesis that the vestibular system is involved in driving and, moreover, that self-reported driving difficulty may be a marker of more severe vestibular dysfunction.

This study builds on previous work suggesting that patients with vestibular disorders may experience driving difficulty. One case-series reported that some patients with vestibular impairments were found making repeated, inappropriate turns in the direction of the imbalance of vestibular tone during driving, while other vestibular patients reported experiencing an illusion that the vehicle was going off course, leading to disorientation, nausea, and panic (28). In a national survey, 44% of adults with bilateral vestibular loss reported that they had either stopped driving or changed their driving habits due to their symptoms (29). Studies of patients with specific vestibular conditions found that 60% of Meniere’s disease patients found driving either difficult or dangerous to perform (17) and 30% of patients with vestibular schwannomas experienced difficulty driving a car following surgical removal of their tumors (20). Certain driving conditions may be particularly challenging for patients with vestibular impairments. One study found that patients with peripheral vestibular disorders report greater difficulty compared to controls in conditions requiring greater spatial navigation ability like pulling into and out of parking spaces and changing lanes in traffic, as well as conditions involving limited visual input such as driving at night or in the rain (19). Studies of on-road behaviors found that bilateral vestibular loss patients have slower horizontal head movements during driving compared to controls (30, 31) and have impaired performance reading and processing signs while riding a car due to abnormalities of the vestibulo-ocular-reflex (32). The current findings build on previous studies of vestibular patients, by demonstrating an association between vestibular dysfunction and driving difficulty in a nationally representative, population-based sample.

The association between vestibular dysfunction and difficulty driving may be a manifestation of the role of the vestibular system in spatial cognition. Visuospatial impairment is known to be a major contributor to unsafe driving in older adults (13). Meanwhile an emerging body of literature suggests that the vestibular system is crucial in spatial orientation and navigation (1, 2, 4, 6, 7, 33). In several recent animal studies, peripheral vestibular ablation in rodents has been shown to lead to impaired performance on tasks of spatial memory and navigation (3, 34–37). Furthermore, imaging studies have found that the hippocampus, which is thought to mediate spatial cognition, is activated by vestibular stimulation (38) and atrophies in patients with bilateral vestibular loss (39). Vestibular input has been further shown to mediate performance on the TCT, a dynamic spatial navigation test (5, 6). Our current results extend previous findings elucidating the role of vestibular function in spatial navigation, by demonstrating a real-world behavioral correlate of the phenomenon. This conclusion is further corroborated by the evidence that vestibular patients report greater difficulty driving particularly during conditions that require spatial navigation, as compared to controls (19).

Although the role of vestibular input in spatial cognition may be the primary mechanism underlying the contribution of vestibular dysfunction to driving difficulty (i.e., *via* vestibulo-cortical pathways), several additional mechanisms have been proposed (e.g., *via* vestibulo-limbic pathways or the vestibulo-ocular reflex) (18). Certain vestibular patients, such as those with Meniere’s disease, may experience attacks of vertigo or dizziness from rapid head movements during driving and subsequently experience anxiety about finding safe places to pull over on the side of the road (17, 19). However, in our current study, participants with asymptomatic vestibular dysfunction also had an increased prevalence of driving difficulty compared to those without vestibular dysfunction, suggesting that episodes of vertigo and dizziness and associated anxiety may not be the most prominent contributing factors. Additionally, it has been suggested that patients with vestibular dysfunction may experience impairments in dynamic visual acuity due to reduced vestibulo-ocular reflex (40), causing difficulty reading stationary signs while in a moving car (32). Given that driving requires the interaction of multiple cognitive domains and sensory inputs and the multiple functions of the vestibular system, it is likely that vestibular dysfunction contributes to driving impairment through more than one distinct mechanism. Further studies will need to examine the contributions of specific dimensions of vestibular function in driving to further elucidate the mechanisms involved.

The vestibular system plays a crucial role in gait stability, balance, and falls risk (41–45). Our finding that vestibular dysfunction attenuates the association between driving difficulty and falls suggests that driving difficulty may serve partially as a marker of severity of vestibular impairment. Interestingly, driving difficulty was found to be an independent contributor to falls risk irrespective of vestibular dysfunction. Driving difficulty may thus reflect other deficits that are not related to vestibular function, such as vision, depression, or deficits in central processing (24, 25, 46). Alternatively, it is possible that the postural metric used in the NHANES to estimate vestibular function does not capture all the dimensions of vestibular function and residual confounding by vestibular function may still be present in the relationship between driving difficulty and falls. A recent study demonstrated a significant relationship between postural sway on condition 4 of the modified Romberg and semicircular canal function but not otolith function (47). It is conceivable that driving difficulty, similar to performance on the TCT, may reflect impairments in both otolith and semicircular canal function (6). Although some evidence has suggested that falls may be a predictor of motor vehicle collisions and changing driving habits among older drivers (26, 27, 48, 49), this study is the first to our knowledge to support the hypothesis that self-reported driving difficulty may be a marker of more severe vestibular dysfunction and serve as a predictor of falls.

We note important limitations of our study. The study was cross-sectional and thus cannot support causal inferences between vestibular dysfunction and driving difficulty. Moreover, the postural metric used in the NHANES to estimate vestibular function is not a specific physiologic test of peripheral vestibular function. Despite this limitation, recent evidence has suggested that performance on condition 4 of the modified Romberg is associated with semicircular canal function as determined by video head impulse testing (47), as well as perceptual thresholds of vestibular functioning (50). Postural tests may be affected by a participant’s strength and musculoskeletal status (e.g., arthritis), as well as by motivation and volitional factors that may affect test compliance (51). However, in this study, these considerations may be mitigated by the fact that participants were only tested in condition 4 if they were able to pass the first three conditions. Moreover, the main finding of this study that performance on the postural metric is associated with driving difficulty further suggests that the postural metric may indeed represent vestibular function, as it is unlikely that factors such as postural control and gait performance are directly associated with driving difficulty. As such, given the technical complexity of vestibular physiological testing, the Romberg on foam with eyes closed test has been put forward as an objective proxy for vestibular function that can be performed on a large sample of participants (52).

In summary, the current study found that vestibular dysfunction was significantly associated with driving difficulty in a nationally representative, population-based sample, while those with both vestibular dysfunction and driving difficulty were at a markedly increased risk of falls. These findings have immediate clinical relevance. Clinicians seeing individuals with vestibular loss should be aware of potential concomitant driving impairments in these patients. Conversations about driving difficulty may present a valuable opportunity to counsel patients on driving habits and transportation alternatives, as driving ability may be an important determinant of quality of life (53–56). Furthermore, patients with vestibular dysfunction who self-report driving difficulty may benefit from early falls prevention intervention such as physical therapy, to reduce the risk of falls and subsequent injuries.

## Ethics Statement

This study was carried out in accordance with the recommendations of National Center for Health Statistics Research Ethics Review Board with written informed consent from all subjects. All subjects gave written informed consent in accordance with the Declaration of Helsinki. The protocol was approved by the National Center for Health Statistics Research Ethics Review Board.

## Author Contributions

EW substantially contributed to the conception and design of the work, analyzed and interpreted the data, drafted the manuscript and revised it critically for important intellectual content, finally approved the version to be published, and agreed to be accountable for the work. YA substantially contributed to the conception and design of the work, analyzed and interpreted the data, revised the work critically for important intellectual content, finally approved the version to be published, and agreed to be accountable for the work.

## Conflict of Interest Statement

The authors declare that the research was conducted in the absence of any commercial or financial relationships that could be construed as a potential conflict of interest.
